# Supplementary figures and images for: MiR-1208 Increases the Sensitivity to Cisplatin by Targeting TBCK in Renal Cancer Cells
Source: Int J Mol Sci. 2019 Jul 19;20(14):3540. doi: 10.3390/ijms20143540 (PMC6679220; doi:10.3390/ijms20143540)

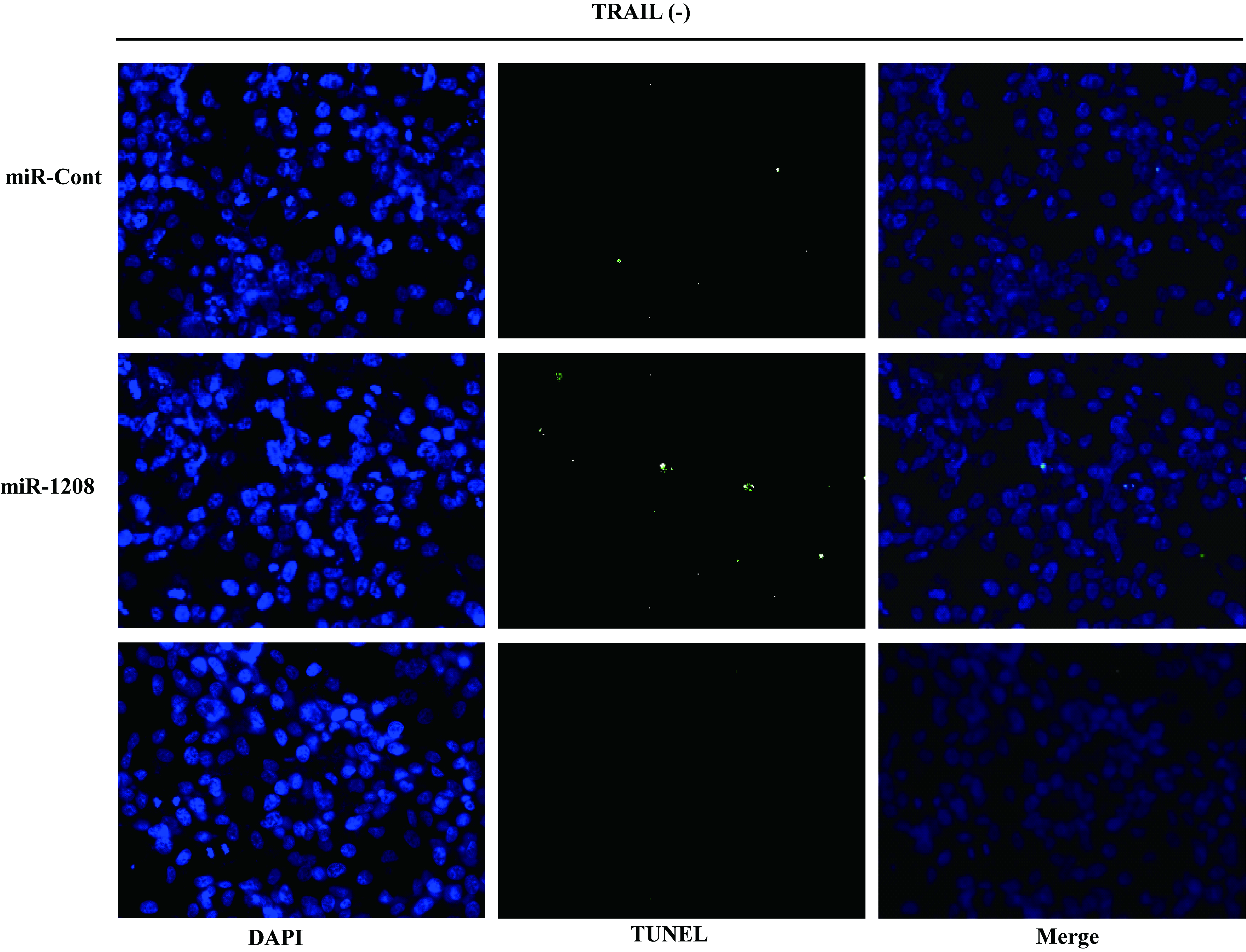

Supplement: Supplementary file 1 [file ijms-20-03540-s001.zip › Suppl 2-1.tif]

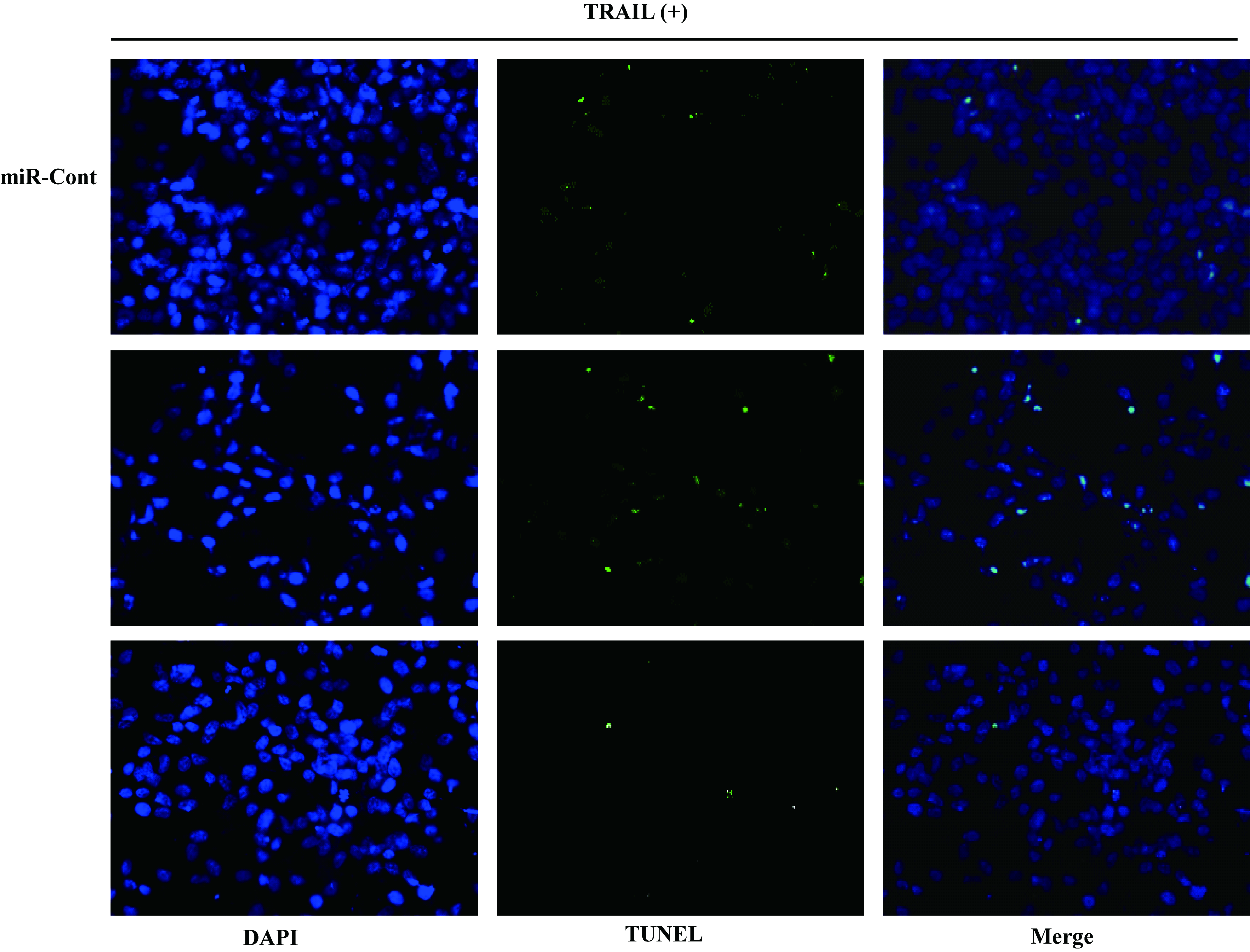

Supplement: Supplementary file 1 [file ijms-20-03540-s001.zip › Suppl 2-2.tif]

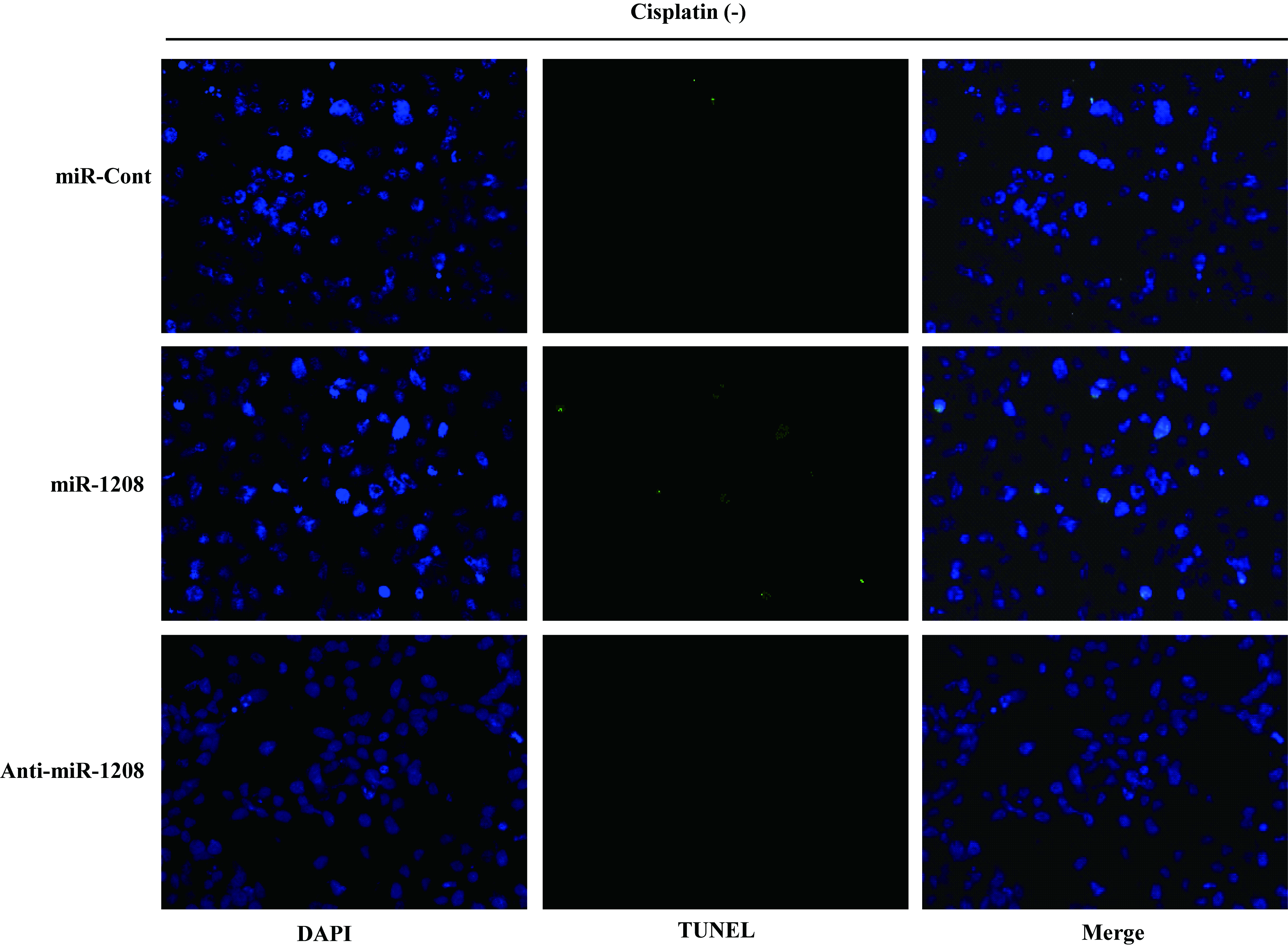

Supplement: Supplementary file 1 [file ijms-20-03540-s001.zip › Suppl1-1.tif]

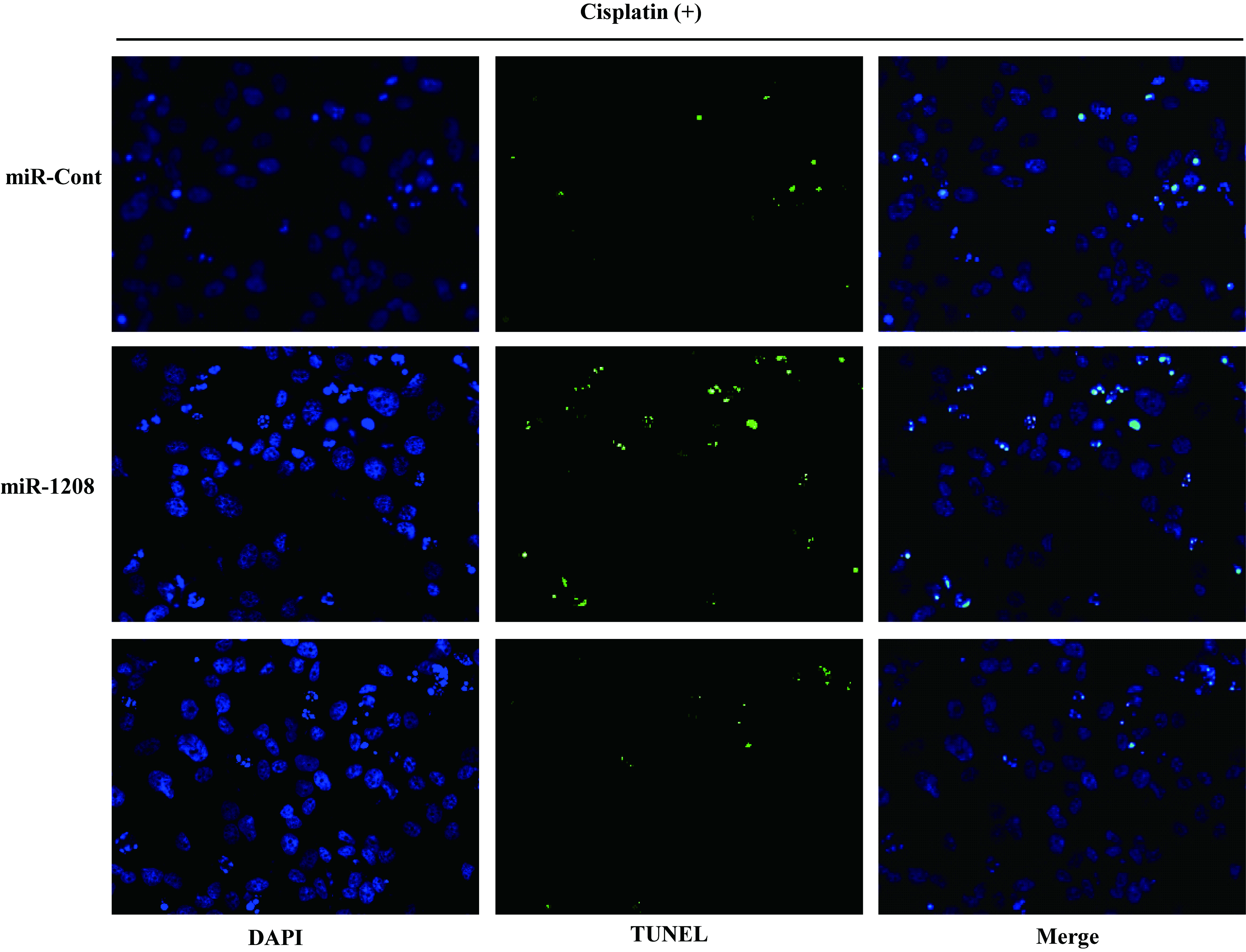

Supplement: Supplementary file 1 [file ijms-20-03540-s001.zip › Suppl1-2.tif]
